# Immune cells lacking Y chromosome show dysregulation of autosomal gene expression

**DOI:** 10.1007/s00018-021-03822-w

**Published:** 2021-04-10

**Authors:** Jan P. Dumanski, Jonatan Halvardson, Hanna Davies, Edyta Rychlicka-Buniowska, Jonas Mattisson, Behrooz Torabi Moghadam, Noemi Nagy, Kazimierz Węglarczyk, Karolina Bukowska-Strakova, Marcus Danielsson, Paweł Olszewski, Arkadiusz Piotrowski, Erin Oerton, Aleksandra Ambicka, Marcin Przewoźnik, Łukasz Bełch, Tomasz Grodzicki, Piotr L. Chłosta, Stefan Imreh, Vilmantas Giedraitis, Lena Kilander, Jessica Nordlund, Adam Ameur, Ulf Gyllensten, Åsa Johansson, Alicja Józkowicz, Maciej Siedlar, Alicja Klich-Rączka, Janusz Jaszczyński, Stefan Enroth, Jarosław Baran, Martin Ingelsson, John R. B. Perry, Janusz Ryś, Lars A. Forsberg

**Affiliations:** 1 Department of Immunology, Genetics and Pathology and Science for Life Laboratory, Uppsala University, Uppsala, Sweden; 2 Faculty of Pharmacy and 3P Medicine Laboratory, International Research Agendas Programme, Medical University of Gdańsk, Gdańsk, Poland; 3 International Research Agendas Programme, 3P Medicine Laboratory, Medical University of Gdańsk, Gdańsk, Poland; 4 Department of Cell and Molecular Biology, Karolinska Institutet, Stockholm, Sweden; 5 Department of Clinical Immunology, Institute of Paediatrics, Jagiellonian University, Collegium Medicum, Kraków, Poland; 6 MRC Epidemiology Unit, School of Clinical Medicine, University of Cambridge, Cambridge, UK; 7 Department of Tumour Pathology, Kraków Branch, Maria Skłodowska-Curie Memorial Cancer Centre and Institute of Oncology, Kraków, Poland; 8 Department and Clinic of Urology, Jagiellonian University, Collegium Medicum, Kraków, Poland; 9 Department and Clinic of Internal Medicine and Gerontology, Jagiellonian University, Collegium Medicum, Kraków, Poland; 10 Department Oncology-Pathology, Karolinska Institutet, Stockholm, Sweden; 11 Department of Public Health and Caring Sciences/Geriatrics, Uppsala University, Uppsala, Sweden; 12 Department of Medical Sciences, Uppsala University, Uppsala, Sweden; 13 Department of Medical Biotechnology, Faculty of Biochemistry, Biophysics and Biotechnology, Jagiellonian University, Kraków, Poland; 14 Department of Urology, Maria Skłodowska-Curie Memorial Cancer Centre, Institute of Oncology, Kraków Branch, Kraków, Poland; 15 The Beijer Laboratory, Uppsala University, Uppsala, Sweden

**Keywords:** LOY, Mosaic loss of chromosome Y, Differential gene expression, LATE, LOY-associated transcriptional effects

## Abstract

Epidemiological investigations show that mosaic loss of chromosome Y (LOY) in leukocytes is associated with earlier mortality and morbidity from many diseases in men. LOY is the most common acquired mutation and is associated with aberrant clonal expansion of cells, yet it remains unclear whether this mosaicism exerts a direct physiological effect. We studied DNA and RNA from leukocytes in sorted- and single-cells in vivo and in vitro. DNA analyses of sorted cells showed that men diagnosed with Alzheimer’s disease was primarily affected with LOY in NK cells whereas prostate cancer patients more frequently displayed LOY in CD4 + T cells and granulocytes. Moreover, bulk and single-cell RNA sequencing in leukocytes allowed scoring of LOY from mRNA data and confirmed considerable variation in the rate of LOY across individuals and cell types. LOY-associated transcriptional effect (LATE) was observed in ~ 500 autosomal genes showing dysregulation in leukocytes with LOY. The fraction of LATE genes within specific cell types was substantially larger than the fraction of LATE genes shared between different subsets of leukocytes, suggesting that LOY might have pleiotropic effects. LATE genes are involved in immune functions but also encode proteins with roles in other diverse biological processes. Our findings highlight a surprisingly broad role for chromosome Y, challenging the view of it as a “genetic wasteland”, and support the hypothesis that altered immune function in leukocytes could be a mechanism linking LOY to increased risk for disease.

**Supplementary Information:**

The online version contains supplementary material available at 10.1007/s00018-021-03822-w.

## Introduction

For over 50 years it has been noted that chromosome Y is frequently lost in the leukocytes of aging men [1, 2] representing the most commonly observed form of clonal mosaicism. Our recent work demonstrated that one in five men in the UK Biobank study have detectable LOY in blood [3, 4] reaching a prevalence of 57% in 93-year-old men [5]. Furthermore, LOY is considerably more common in peripheral blood leukocytes vs. other tissues [5, 6]. These observations have been accompanied by epidemiological studies linking LOY in blood to numerous disease outcomes, including all-cause mortality, Alzheimer’s disease, various forms of cancer, autoimmune conditions, age-related macular degeneration, cardiovascular disease, type 2 diabetes, and obesity [6–15]. Associations between LOY and blood cell counts have also been reported; platelet and red blood cell numbers are positively and negatively associated with LOY, respectively [16, 17].

Recent large-scale population studies have highlighted a heritable component to LOY and begun identifying individual genetic determinants. The largest to date, studying 205,011 men in UK Biobank, identified 156 genetic loci associated with LOY, which were preferentially found near genes involved in cell-cycle regulation, cancer susceptibility, somatic drivers of tumor growth, and cancer therapy targets [4]. The emerging picture is that LOY is in part determined by genetic predisposition to deficiencies in DNA damage response—either through genetic effects that promote chromosome mis-segregation or failure in the molecular machinery to detect and appropriately deal with this damage. The remaining part might be due to other risk factors, e.g., smoking and environmental hazards [18–20].

There are two main, not mutually exclusive hypotheses that could explain the link between LOY in blood and risk for disease—either loss of a Y chromosome in leukocytes exerts a direct physiological effect, and/or LOY in leukocytes is a barometer of broader genomic instability in other cell types. Evidence for the latter emerged with the observation that genetic susceptibility to LOY influences non-hematological health outcomes in women (who are XX, hence eliminating a direct Y effect). This observation does not, however, preclude a direct effect of LOY that may explain some of the noted disease associations. Indeed, we recently reported that expression of *TCL1A* was dysregulated in CD19 + B-lymphocytes missing the Y chromosome, providing a proof-of-concept that LOY may not be functionally neutral in these cells [4]. The current study substantially expands this observation, demonstrating that LOY is associated with dysregulation of autosomal gene expression in leukocytes. Furthermore, the biological nature of these dysregulated genes supports the hypothesis that altered immune response may be a mechanism linking LOY in immune cells to disease.

## Materials and methods

### Sample collection

Blood samples were collected from 408 male subjects in Uppsala, Sweden, and in Kraków, Poland. In Uppsala, samples from ULSAM, Alzheimer’s disease cohort (UAD), and controls (M) were collected during January 2015 to May 2018, at the Geriatric/Memory Clinic, Uppsala Academic Hospital. In Kraków, samples from prostate cancer patients (KP) were collected from March 2015 to May 2018 at the Centre of Oncology, Kraków Branch and the Department and Clinic of Urology of the Jagiellonian University. Alzheimer’s disease (KAD) patients were collected from January 2017 to May 2018 at the Clinic of Internal Diseases and Gerontology of the Jagiellonian University in Kraków. Control samples (M) were collected from December 2015 to May 2018 from the general population of Kraków and Uppsala. The criteria for recruitment of prostate cancer patients was advanced stage prostate cancer, i.e., Gleason grade 7 or higher. The prostate cancer patients were recruited before treatment, or during the first stage of treatment. As for Alzheimer’s disease, patients with ongoing clinically and radiologically confirmed diagnosis were recruited. The clinical stage for recruited Alzheimer’s patients was intermediate or severely advanced disease. For the purpose of LOY analysis in sorted leukocyte subsets (Fig. 3), we used 107 prostate cancer patients, 121 Alzheimer’s disease patients, and 156 age-matched controls.

### Preparation and sorting of blood cells with FACS

We implemented two strategies for preparation of blood cells for sorting.

#### Isolation and labeling of peripheral blood mononuclear cells (PBMCs) for sorting of T- and B-lymphocytes as well as NK cells

16 ml of blood was collected into BD Vacutainer® CPT™ Mononuclear Cell Preparation Tubes (BD). PBMCs were stained with 20 µl of BD Multitest™ 6-color TBNK reagent (BD) and incubated for 20 min. at 4 °C. After incubation, PBMCs were washed with PBS and cell pellets were resuspended in 1 ml of PBS containing 3 mM EDTA. PBMCs were filtered through 40 µm cell strainer to obtain real single cell suspensions by removing cell aggregates.

#### Isolation and labeling of white blood cells (WBCs) for sorting of granulocytes, monocytes, and B-lymphocytes

16 ml of blood was collected into BD Vacutainer® K2 EDTA tubes (BD). Red blood cells were lysed using 1 × BD Pharm Lyse™ lysing solution (BD) added to blood samples up to 50 ml. Samples were incubated at room temperature for 10 min. The lysing step was repeated and cells were then washed with PBS. Isolated white blood cells (WBCs) were stained with following antibodies: 5 µl of PE-labeled CD14, clone MφP9 (BD), and 20 µl of APC-labeled CD19, clone HIB19 (BD) and incubated for 20 min. at 4 °C. After incubation, WBCs were washed with PBS and cell pellets were resuspended in 2 ml of PBS containing 3 mM EDTA.

#### Sorting of target cell populations with FACS

The target populations were isolated using FACS Aria III (Becton Dickinson), FACS Aria II (Becton Dickinson), and MoFlo (Beckman Coulter). Data were acquired and analyzed using BD FACSDiva™ Software (Becton Dickinson) with FACS Aria III and FACS Aria II or Summit™ Software System (Cytomation) with MoFlo. Live cells were sorted based on their FSC and SSC. CD4 + T cells were defined as CD45 + CD3 + CD8 − CD4 + ; CD8 + T cells were defined as CD45 + CD3 + CD4 − CD8 + ; B cells were defined as CD45 + CD3 − CD19 + ; NK cells were defined as CD45 + CD3 − CD4 − CD16 + CD56 + ; monocytes were defined based on their size and as CD14 + ; granulocytes were defined based on their size and granularity, and additional B cells were defined based on their size and CD19 + . Cells were sorted to achieve the purity of above 96%. At least 200,000 cells of each type were sorted. After sorting, cells were centrifuged and 1 ml of RNAProtect (Qiagen) was added to cell pellets.

### Establishment of lymphoblastoid cell lines (LCLs)

The LCLs were generated using Epstein–Barr virus (EBV) transformation of lymphocytes collected from six male donors with previously established LOY in whole blood [5]. These control subjects were derived from the Uppsala Longitudinal Study of Adult Men (ULSAM) cohort and were free from cancer or Alzheimer’s disease diagnosis at the age of 91–94 years. Peripheral blood mononuclear cells (PBMC) were isolated from blood by Ficoll-Paque gradient separation. PBMC were infected with EBV by incubating them with supernatant of the virus producing B95-8 line for 90 min at 37 °C. Thereafter, the cells were washed and resuspended in complete RPMI-1640 medium (supplemented with 10% heat inactivated FCS, penicillin, and streptomycin). 1 µg/ml Cyclosporine A was added to the cultures to inhibit T cell expansion and function. After 3 weeks of culturing, transformed cells were plated into microtitre plates at a 1 cell/well density. In each well, gamma-irradiated human fibroblasts (5000 rad) served as feeder cells.

### LOY analyses using DNA from sorted cells and LCLs

#### DNA extraction

DNA was extracted using an in-house protocol with lysis buffer containing 10 mM EDTA, 10 mM Tris–HCL (pH 7.9), 50 mM NaCl, and 1% N-Lauroylsarcosine sodium salt (Sigma) with 10 mg/ml proteinase K (Sigma) and incubated for 2 h in 50 °C. The DNA was then precipitated and resuspended in water. DNA was extracted from blood using QIAmp DNA Blood Midi kit (Qiagen). The concentration was measured using Quant-iT™ PicoGreen® dsDNA Assay Kit (Thermo Fisher Scientific).

#### Genotyping and LOY analysis using SNP-arrays

All genotyping experiments were performed following the manufacturer’s instructions at the Science for Life technology platform SNP&SEQ at Uppsala University, Sweden. DNA extracted from the FACS sorted populations was genotyped using three different versions of Illumina SNP-arrays (InfiniumCoreExome-24v1-1, InfiniumOmniExpressExome-8v1-3 and InfiniumQCArray-24v1). All included experiments passed strict quality control at the genotyping facility and additional quality assessment as described previously [7]. The Log R Ratio (LRR) is a commonly used metric to determine copy number states from SNP-array data. The “R” represents probe intensity estimates and the LRR is the logged ratio of the observed probe intensity to the expected intensity. Thus, deviations from zero in the LRR values of probes located in a specific genomic region is indicative of copy number change. For the estimation of LOY mosaicism in the present study, we calculated the median of the LRR values (i.e., the mLRRY) of the SNP-array probes located in the male-specific region of chromosome Y (MSY) as described previously [7]. This generated a continuous estimate of LOY for each sample. Thus, mLRRY estimates around zero indicate a normal chromosome Y state, whereas samples with higher levels of LOY mosaicism show more negative mLRRY values. To correct for genotyping batch effects, we calculated the local regression median (LRM) in each batch and the mLRRY value for each subject was, thereafter, corrected by the batch-specific LRM. The percentage of normal cells without LOY in each sample was estimated using a formula described previously, i.e., using the formula: LOY(%) = 100*(2^2*mLRRY^) [21].

#### Experiments and LOY analysis using digital droplet PCR (ddPCR)

Additional quantification of the level of LOY for validation was performed using previously described procedure [21]. Briefly, extracted DNA was digested for 15 min with HindIII enzyme (Thermo Fischer) in 37 °C. After this, 50 ng of DNA was added together with PCR primers and probes targeting a known difference between the *AMELX* and *AMELY* gene assay C_990000001_10 (Thermo Fisher). Droplets were then generated using an automated droplet generator (Bio-Rad) and amplified using PCR. Droplets fluorescence intensity in two channels FAM and VIC was measured using the Bio-Rad’s QX200 Droplet Reader. The data were analyzed in Bio-Rad’s software QuantaSoft (version 1.7.4.0917) as described elsewhere [21]. The LOY quantification was performed by calculating the ratio between the amounts of *AMELY* and *AMELX* in each sample.

### Experiments using bulk RNA from sorted cells and LCLs

#### RNA extraction

RNA was extracted from cell pellets using RiboPure™ RNA Purification Kit (Thermo Fisher Scientific) according to the manufacturer’s instructions. Possible DNA contamination was removed using TURBO DNA-free™ Kit (Thermo Fisher Scientific). The RNA was then concentrated using GeneJET RNA Cleanup and Concentration Micro Kit (Thermo Fisher Scientific). RNA quality was measured with RNA Pico Chip (Agilent Technologies) and analyzed using Agilent 2100 Bioanalyzer (Agilent Technologies).

#### Bulk RNA sequencing (RNAseq)

Bulk RNA sequencing was performed by the Science for Life technology platform at Uppsala Genome Centre (Uppsala University, Sweden). For each sample, an average of 10 ng of RNA (1–100 ng) from the FACS sorted samples was used. Library preparation was performed using the Ion Ampliseq Human Gene Expression kit (Thermo Fisher Man0010742). Libraries were loaded onto Ion 550 chips following recommendations from the manufacturer (MAN0017275) and sequenced using an Ion S5 XL instrument.

#### Analysis pipeline of bulk RNAseq data from sorted cells

For each of the FACS sorted samples analyzed using the Ion Ampliseq Human Gene Expression kit, raw read-counts were read into the R (v3.5.3) software, and one matrix of read-counts was created per cell type. Outliers and low quality expression data were identified within each cell type using principal component plots. Only among the monocyte samples, eight outliers were identified and removed. Estimation of the level of LOY in each sample in the RNAseq data was performed by a variance-stabilizing transformation applied in the R library DESeq2 (v1.22.2) [22] using the varianceStabilizingTransformation function, to the count matrix to make the expression more coherent. A mean expression was then calculated for all genes on the autosomal chromosomes, and compared to the mean expression of the MSY genes, generating a fraction of expression for the Y chromosome. The calculated fractions were rescaled to fit between 0 and 100. This was done per cell type, to avoid cell type specific biases in expression levels. Next, normally expressed genes in the sorted cell fractions were identified by the analysis of samples without LOY. In these, genes with at least ten sequencing reads in more than 75% of the samples without LOY were considered normally expressed. To investigate associations between LOY and expression level of chromosome Y genes, we applied a non-linear least square model, using nls() function in R, on average expression of all selected MSY and PAR genes and percentage of LOY in samples of each cell type. For each cell type, the expression level of each sample was divided by the maximum expression level among all samples to generate estimates between 0 and 1. We estimated the LOY-associated transcriptional effects (LATE) in the RNAseq data as follows. DESeq2 (v1.22.2) [22] was used to identify genes with differential expression in the leukocytes derived from each patient. As LOY is a continuous trait, the percentage of LOY estimated from SNP-arrays for each sample was used for statistical testing. To decrease the impact of read-count outliers the fraction of LOY was binned using 20% intervals between < 20% and 80% < and these bins were used as a continuous input variable for DESeq2. We investigated the effect on gene expression from known and potentially confounding variables (e.g., age, clinical characteristics, collection center, and sequencing batch) and determined to include sequencing batch as a covariate in the analysis of differentially expressed genes. Independent filtering of genes in DESeq2 was done using the default value of 0.1 for the alpha parameter, which was also used as a cutoff for the DESeq2 adjusted *p* values.

#### Analysis pipeline of bulk RNAseq data from LCLs

We used R (version 3.5.2) and the edgeR (version 3.24.3) library to compare the expression between LCLs with the Y chromosome and LCLs with LOY in two steps. As all the clones with LOY were derived from the same individual (this individual also gave rise to one non-LOY clone), we designed the analysis to remove inter- and intra-individual differential expression effects. As step one, to identify the intra-individual differences, we targeted the differential expression genes among the normal LCLs by comparing each non-LOY individual’s clones with all other non-LOY clones. The union of all differentially expressed genes resulted out of each comparison was gathered into List1 (Supplementary Fig. 5). This gave us a set of genes that are differentially expressed between subjects but not due to LOY status. As step two, we targeted the differentially expressed genes regarding LOY status in two steps: first by comparing the non-LOY versus LOY clones derived from the same individual; second by comparing all non-LOY versus LOY clones and the overlap of the results of steps one and two was gathered into List2. The final list of LATE genes was generated by comparing List2 and List1 and extracting the differentially expressed genes that exist in List2 but not in List1, to remove the intra-individual differentially expressed genes from the LATE genes.

### Experiments using RNA from single cells

#### Sample preparation

Blood was collected using BD Vacutainer CPT tubes (BD Biosciences). PBMCs were isolated using density gradient centrifugation and resuspended in 0.04% BSA 1X PBS solution. Concentrations were measured using an EVE cell counter (NanoEnTek, Seoul) and cells were diluted to 10^6^ cells/ml.

#### Single cell RNA sequencing (scRNAseq)

The collected PBMCs were loaded on a 10X Genomics Chromium Single Cell 3′ Chip v2 for sequence library generation using protocol CG00052 (10X Genomics) using a 3′-end protocol. The single cell libraries were sequenced on Illumina HiSeq2500 and NovaSeq instruments The single cell library preparation and sequencing were performed at the Science for Life technology platform SNP&SEQ, Uppsala University, Sweden.

#### Analysis pipeline of single cell scRNAseq data

Sequence reads were mapped to the hg19 version of the human genome using CellRanger v2 (10X Genomics) and recommended settings. The produced raw reads matrices were analyzed using R (v3.4) and the R library Seurat (v2.2) [23]. For each sample, the read-count matrix was used to create a Seurat object in R. After this, all data were log normalized and variable genes were identified using standard settings from the Seurat manual. Data were then scaled and the principal components were calculated. Then, the number of informative principle components for each sample was identified using an elbow plot and cells were then clustered using the findclusters Seurat function and informative principle components. A tSNE plot was produced with the resulting clusters of the nearest neighbor clustering indicated by the color of each cell in the tSNE plot, to confirm consistency. An in-house R script was then used to calculate the level of expression of cell marker genes in each cluster and cell types was assigned using the strongest expression signal. These results were then manually inspected using heatmaps to confirm the automated cell-type assignment. Following this step, all individuals were merged together creating one object for each studied cell type (monocytes, NK cells, T- and B- lymphocytes). The procedure outlined above was repeated for the merged data sets and the cells were filtered only keeping cells with at least 800 expressed genes but no more than 2000 expressed genes. This was done both to remove experiments where two cells were sequenced together, and to remove cells with a generally low expression of genes, where the LOY estimate may be erroneous. Cells with more than 5% mitochondrial RNA were also excluded, to remove possibly apoptotic and damaged cells from the analysis. To determine the LOY status in each single cell the number of reads mapping to autosomal genes and genes located in the male-specific region of chromosome Y (MSY) was counted. Each sequenced cell with expression of autosomal genes, but without any transcripts from genes located in the MSY was considered as a LOY cell as described elsewhere [4]. To calculate the percentage of LOY in each sample and cell types, the fraction of cells having LOY was calculated for each specific cell type. Normally expressed genes were defined by the expression in at least 10% of single cells without LOY. After establishing the normally expressed genes in each cell type in the scRNAseq data, we further filtered the cells to include only cells having more than 2500 UMIs. This was done to remove possible false positive LOY cells, and only keep cells where a robust and high number of UMIs was found. We then identified genes showing differential expression (i.e., LATE genes) in the single cells dataset using the Seurat FindMarkers function. We used this function to test differences in gene expression in cells with and without LOY using the built-in negative binomial test.

#### Co-expression analysis using single cell RNA dataset

We investigated if changes in expression as an effect of LOY would be larger in autosomal genes that are normally co-expressed with MSY genes, compared with genes that are not co-expressed with MSY genes. We first identified six MSY genes that showed expression in normal leukocytes (i.e., without LOY) in the single cell data set (i.e., *RPS4Y1*, *ZFY*, *USP9Y*, *DDX3Y*, *KDM5D*, and *EIF1AY*). Next we identified the degree of co-expression between these six MSY genes and autosomal genes using the default settings in the SEEK database (http://seek.princeton.edu/). The applied algorithm identifies genes showing similar expression patterns across human samples in a large number of databases containing gene expression data from numerous sources [24]. From the generated list, we selected the top 300 autosomal genes showing the most co-expression with the MSY genes and calculated the fold change of the changes in expression of these 300 genes in single cells with LOY compared with normal cells without LOY. Additionally, we calculated the equivalent fold changes for autosomal genes not showing co-expression with MSY genes. Following this, the fold changes for the co-expressed and the not co-expressed sets of autosomal genes was used to produce plots, and a Wilcoxon rank sum test was used to test for differences. Similar analyses were also performed separately within monocytes, NK cells, T- and B-lymphocytes, in which 142, 116, 95, and 122 co-expressed autosomal genes as well as 3677, 3230, 2752, and 3075 autosomal control genes without co-expression were identified, respectively.

### Functional annotation of chromosome Y genes

For annotation of known functions of chromosome Y genes summarized in Supplementary Fig. 1, a list of all genes located on the Y chromosome together with associated GO categories was downloaded from Ensembl (v. 95) using the R library bioMart (v2.38). The list was filtered for protein coding genes and annotated as either positioned in the male-specific part of the Y chromosome (MSY) or in the pseudo-autosomal regions (PAR). The GO categories for each gene were inspected manually and genes were assigned into four different categories: “*Transcription, translation epigenetic, and PTM functions*”, “*Cell differentiation, migration proliferation, and apoptosis*”, “*Sex determination, fertility, and other functions*”, and “*Immune response and immune cell signaling*”.

### Non-random distribution of LATE genes

Over-representation of known biological connections between genes was calculated using the STRING web tool [25]. To evaluate the genomic clustering of LATE genes we plotted the largest differential expression (Supplementary Table 1) of each of the 489 autosomal LATE genes using a modified version of the plotManhattan function from the R package qqman [26] with chromosome lengths from https://www.ncbi.nlm.nih.gov/grc/human/data (assembly GRCh37).

### Enrichment testing of LOY GWAS variants

To test the hypothesis that LATE genes might be preferentially located near LOY-associated variants highlighted by GWAS [4] we performed gene set enrichment analysis using MAGENTA [27]. This was performed with the full GWAS summary statistic data using the recommended default running parameters of MAGENTA.

### Gene set enrichment analyses

The 489 autosomal LATE genes were compared to the background of 11,884 tested genes. Gene symbols were mapped to Entrez Gene IDs using the org.Hs.eg.db Bioconductor package [28] resulting in 460 and 10,989 identifiers, respectively. Enrichment of LATE gene Entrez IDs in GO terms was obtained as a DAVID Functional Annotation Chart of GO FAT terms via RDAVIDWebService [29]. Enrichment in Reactome pathways and the GO biological process complete set were additionally performed using the PANTHER [30] overrepresentation test, using Fisher’s exact test statistic.

### Other statistical analyses

Statistical analyses such as logistic regression, linear regression, Fisher exact test, Kolmogorov–Smirnov test, and Wilcoxon rank sum test were performed using R version 3.3.1 (http://www.rproject.org/), using two-sided tests and *α*-levels at 0.05 and default parameters, unless otherwise specified. When appropriate, correction of *p* values for multiple testing was applied. To define the LATE genes, we applied three stringency levels. The first was based on standard *p* value at *α*-level 0.05 and the second was a more stringent *p* value correcting for multiple testing (FDR < 0.1) using Benjamini and Hochberg (BH) correction. Furthermore, an additional criterion was applied to identify only the highly expressed LATE genes, i.e., using threshold of at least an average of 100 reads per gene in RNAseq data.

## Results

To investigate the functional consequences of LOY, we studied changes in gene expression associated with LOY in vivo and in vitro (see “Materials and methods”). The single-cell transcriptome analysis (scRNAseq) of peripheral blood mononuclear cells (PBMCs) collected from 29 men (median age 80 years, range 64–94 years) generated a pooled dataset encompassing 73,606 cells. The Y chromosome contains 64 protein coding genes: 45 in the male-specific region (MSY) and 19 in the pseudo-autosomal regions (PARs). We detected the normal expression of 20 protein coding genes on chromosome Y across all white blood cells (Supplementary Fig. 1), enabling the classification of single cells with or without LOY. We identified single cells with LOY in all 29 subjects studied with scRNAseq and observed that the distribution of LOY varied substantially between cell types (Supplementary Fig. 2). Specifically, the frequency of LOY in NK cells, monocytes, B- and T lymphocytes were 27% (7–87%), 23% (7–87%), 7% (2–40%), and 3% (1–6%), respectively. We also performed bulk RNA sequencing (RNAseq) on 134 samples of sorted cell fractions (NK cells, monocytes and granulocytes) collected from 51 subjects, including the 29 subjects studied with scRNAseq (Supplementary Fig. 2).

We observed a high concordance in LOY estimates generated from pairwise samples studied in vivo with scRNAseq, RNAseq, and DNA based technologies (Supplementary Fig. 3). Furthermore, we studied in vitro changes of gene expression using RNAseq in 13 lymphoblastoid cell lines (LCLs) with or without LOY (Supplementary Figs. 4 and 5). Concordant levels of LOY were observed in LCLs studied with SNP-array as well as with a new method for LOY analysis [21] using droplet digital PCR targeting a 6-bp sequence difference between the *AMELY* and *AMELX* genes (Pearson’s correlation coefficient = 0.9961; details not shown).

### Many autosomal genes are dysregulated in LOY cells

**Fig. 1:**
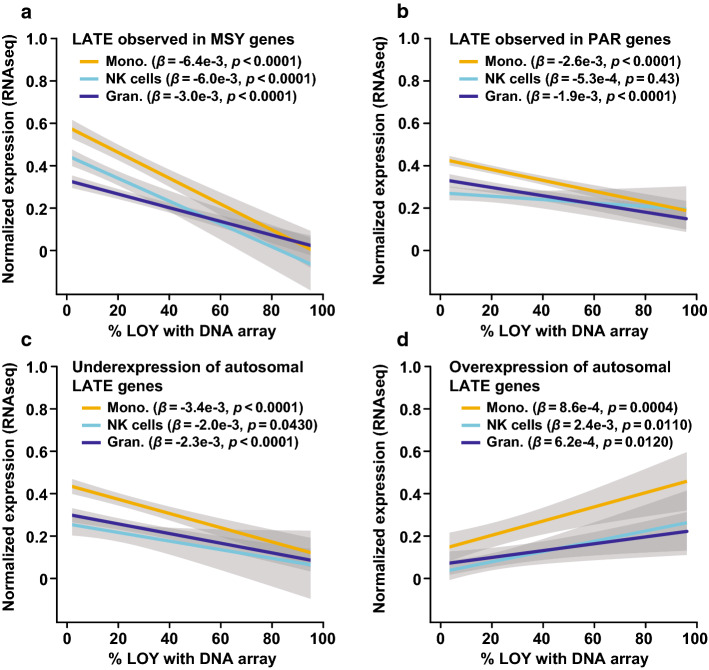
LOY associated transcriptional effect (LATE) in leukocytes in vivo. On the *X*-axes are the level of LOY mosaicism (estimated from DNA by SNP-array from three sorted cell populations) and on the *Y*-axes are the normalized level of gene expression estimated from the same samples using RNAseq. Panel **a** display the average expression of six genes located in the male-specific part of chromosome Y (MSY) as a function of LOY. Panel **b** shows corresponding analysis of 13 genes located in the pseudo-autosomal regions of chromosomes X and Y (PAR). Panels **c**, **d** illustrate the finding of autosomal LATE genes, i.e., genes located on other chromosomes showing reduced or increased abundance of transcripts in samples with LOY. Panel **c** displays the average expression of the ten most underexpressed autosomal LATE genes and the ten most over-expressed autosomal LATE genes is shown in panel **d**. Grey areas represent the standard error of linear regression models and beta (*β*) with confidence estimate (*p*) is shown. *Mono.* monocytes, *Gran.* granulocytes

To identify LOY Associated Transcriptional Effects (LATE), we tested for differential gene expression between the LOY and non-LOY cellular populations (Fig. 1, Table 1, Supplementary Fig. 6, see “Materials and methods”). As expected, MSY gene expression was greatly decreased in the bulk RNAseq on sorted cells and absent in the single cell data (as this was the LOY definition for single cells). A corresponding analysis of the genes located in the PARs also showed a decrease in transcript abundance with increasing levels of LOY. However, the decrease in PAR genes was not as distinct as for the MSY genes, which was expected because of sustained expression of the chromosome X-copy (Fig. 1, Supplementary Fig. 4).

**Table 1:** Number of autosomal genes showing LATE in samples studied by two independent RNA sequencing methods

Cell type	Results from RNAseq analysis^*^		Results from scRNAseq analysis^*^	
	*N*. of expressed genes in samples without LOY	*N*. of LATE genes in samples with LOY	*N*. of expressed genes in normal non-LOY cells	*N*. of LATE genes in single cells with LOY
NK cells	9974	298	3021	37
Monocytes	9891	80	3634	28
Granulocytes	7497	57	–	–
T lymphocytes	–	–	2798	13
B lymphocytes	–	–	3112	3
LCL’s	9549	757	–	–
Number of genes^#^	11,472	1155	4631	75
Number of LATE genes in vivo^#^		420		75

Number of autosomal LATE genes detected in vivo by RNAseq and/or scRNAseq^#^ = 489

^*^Results from RNAseq and scRNAseq analyses are shown separately for each cell type. The detected number (*N*.) of expressed genes as well as the number of LATE genes detected in different cell types using FDR < 0.1 are shown

^#^The number of unique genes after exclusion of overlaps between cell types and/or methods

Outside of the Y chromosome, we found evidence for 489 autosomal LATE genes and 10 on the non-PAR X chromosome across the two expression datasets and all types of leukocytes, from in vivo studied samples (Fig. 1, Table 1, Supplementary Table 1). Of these, 75 were identified in the single cell data and 420 in the bulk RNAseq and we observed overexpression as well as underexpression of specific autosomal LATE genes. The autosomal genes showing the largest LATE were *LYPD2* and *IL1R2*, displaying 8.6 higher (DE = 3.11, FDR = 0.0011) and 2.5 lower (DE = − 1.30, FDR = 0.0612) abundance of transcripts, respectively. A considerable number of genes were independently identified as showing LATE in different cell types and by both technologies (Supplementary Table 2). Co-expression analysis showed that autosomal genes that are normally co-expressed with MSY genes displayed a higher level of differential expression in single cells with LOY compared with control genes (Supplementary Fig. 4, Wilcoxon rank sum test: *p* = 0.0021, see “Materials and methods”). Results from in vitro studied LCL’s further support that LOY is associated with altered autosomal gene expression using a complementary approach (Supplementary Figs. S4 and S5, Kolmogorov–Smirnov test: D = 1.0, *p* = 0.0016).

**Fig. 2:**
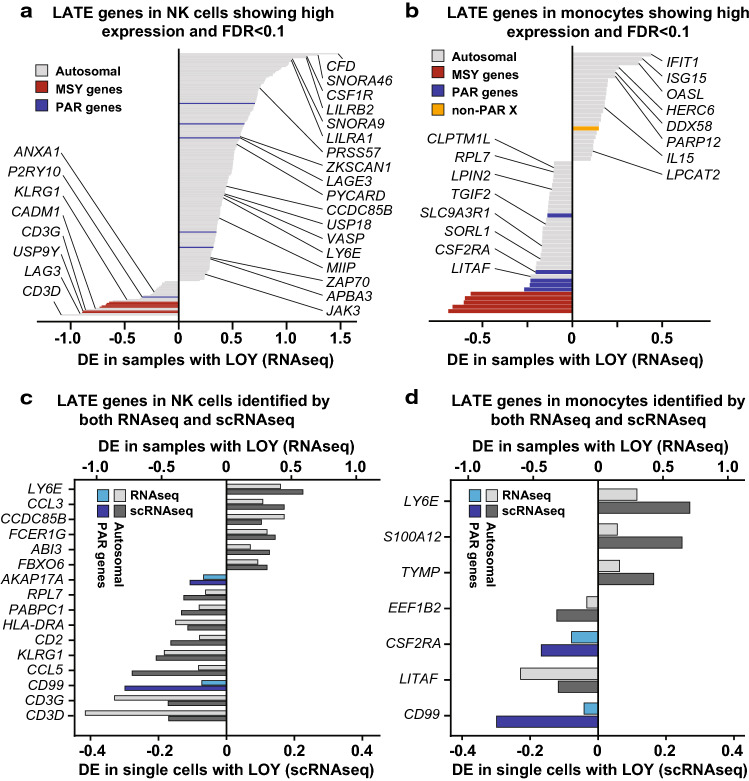
Differential expression (DE) of specific genes as an effect of LOY in NK cells and monocytes, studied by both RNAseq and scRNAseq. Panels **a**, **b** illustrate the level of DE in 206 and 60 autosomal LATE genes identified in each cell type after correction for multiple testing (FDR < 0.1) and with at least an average of 100 reads per gene in samples without LOY. Names are shown for LATE genes known to be linked with immune system functions and/or cancer and/or development of Alzheimer's disease. Panels **c**, **d** display DE observed in autosomal and PAR genes that was identified as LATE genes by both RNAseq and scRNAseq at a 0.05 *α*-level. MSY genes were excluded because of lack of expression in cells without chromosome Y. Independent identification of LATE was observed for 16 and 7 genes in the NK cells and monocytes, respectively. The autosomal gene *LY6E* and the PAR gene *CD99* displayed LATE in both cell types and by both technologies

In the monocytes and NK cells that were studied by two RNA sequencing methods, the identified LATE genes showed an overall concordance in the direction of change in gene expression between technologies (binomial sign test; monocytes: *p* = 0.030, NK cells: *p* = 0.001). The level and direction of differential expression of LATE genes detected independently by both technologies are illustrated in Fig. 2. Among these, two genes were detected in both cell types and by both methods, i.e., upregulation of the *LY6E* gene located on chromosome 8 and downregulation of the PAR gene *CD99*.

Furthermore, we observed that the fraction of LATE genes within specific cell types was substantially larger than the fraction of LATE genes shared between different subsets of cells, in both the RNAseq dataset (ANOVA: *F*_*1,8*_ = 95.5, *p* < 0.0001) and in the scRNAseq dataset (ANOVA: *F*_*1,8*_ = 13.7, *p* = 0.0061). For instance, up to 15% of normally expressed genes showed LATE in NK cells, but only about 2% of LATE genes were shared between NK cells and granulocytes or monocytes (Supplementary Fig. 7). This result emphasizes that transcriptomic alterations associated with LOY vary between different types of immune cells, likely because each cell type normally express a set of genes that is only partially overlapping with the gene expression of other cell types.

### Biological pathways implicated by identified LATE genes

The 489 autosomal LATE genes had twice as many (*p* < 1 × 10^–16^) biological connections amongst each other than expected by chance for a matched randomly selected set of genes. Furthermore, LATE genes were not randomly distributed throughout the genome, with clusters of genes evident (Supplementary Fig. 8). This was most pronounced in the 11q13.1 region, where we identified 17 LATE genes (13 over-expressed in LOY) in an 8 Mb genomic window (Supplementary Table 1). To explore the potential biological relevance of the identified genes, we next performed pathway enrichment analyses, controlling for the background set of all genes expressed in our tested cell types. This resulted in the identification of a large amount of significantly over-represented (FDR < 0.05) GO categories and pathways, the majority of which focused on aspects of RNA processing, immune function and the viral life cycle. Specifically, 191 pathways and GO categories was identified using the DAVID functional annotation tools and 104 was identified using the Panther classification system (Supplementary Table 3). A summary of our literature review for detected LATE genes is presented in Supplementary Table 4.

We next tested whether LATE genes were preferentially located near genetic variants associated with LOY [4]. Gene set enrichment analysis implemented in MAGENTA demonstrated a modest excess of such associations (1.17 fold enrichment, *p* = 0.010), with 41 LATE genes mapping within 300 kb of a genome-wide significant SNP association in 25 genomic regions. In several instances, the proximal LATE gene was the same as that prioritized through gene mapping approaches in the GWAS study. For example, LATE gene cyclin-dependent kinase inhibitor 1C (*CDKN1C*) was linked via expression changes to a nearby LOY-associated variant (rs60808706). Here, the LOY risk increasing allele increases expression of *CDKN1C* and the expression of *CDKN1C* is increased in cells with LOY. As with our previously reported example of *TCL1A* [4], this observation raises the possibility of a bi-directional relationship between LOY and *CDKN1C—*genetically increased expression of the gene promotes LOY and LOY subsequently further dysregulates gene expression in a cell-specific manner, possibly leading to a negative spiral of increased clonal mosaicism via perturbed cell-cycle processes.

### LOY in sorted leukocytes from patients with prostate cancer and Alzheimer’s disease

**Fig. 3:**
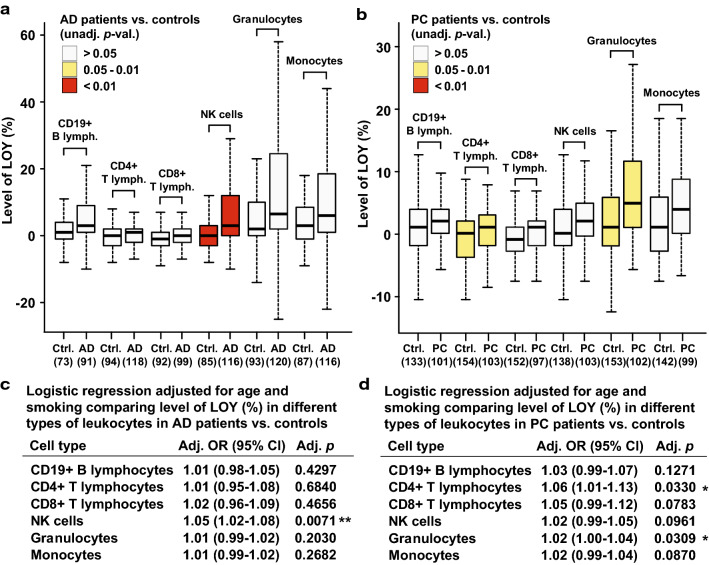
Distribution of LOY in six populations of leukocytes in men diagnosed with Alzheimer’s disease or prostate cancer vs. controls. Leukocytes were sorted by FACS, followed by SNP-array genotyping for each cell fraction and calculation of the percentage of LOY. The numbers in parentheses under the X-axes in panels **a**, **b** denote the number of studied subjects for each cell type. Panels **a**, **b** show results from unadjusted analyses and panels c and d show results from logistic regression models adjusted for age and smoking. *Ctrl.* control, *AD* Alzheimer’s disease, *PC* prostate cancer, *LR* logistic regression, *OR* odds ratio, *lymph.* Lymphocytes, *NK* natural killer

Finally, we investigated the distribution of LOY in six types of sorted leukocytes—CD19 + B lymphocytes, CD4 + T lymphocytes, CD8 + T-cytotoxic lymphocytes, Natural Killer (NK) cells, granulocytes, and monocytes—among men diagnosed with Alzheimer’s disease (*N* = 121) or prostate cancer (*N* = 107) vs. healthy controls (*N* = 156). Overall, there was a general trend for LOY to be increased in patients vs. controls, and this rate varied considerably between cell types (Fig. 3). Men with Alzheimer’s disease had significantly higher levels of LOY only in CD16 + /CD56 + NK cells compared to controls (*p* = 0.0071). The fraction of Alzheimer’s disease cases with high levels of LOY in NK cells was about four times larger compared with controls and supports previous observations that LOY is associated with incident Alzheimer’s disease [9]. Furthermore, the involvement of NK cells in the pathogenesis of Alzheimer’s disease has been suggested before in a range of experimental study designs [31–39]. In prostate cancer patients, NK cells did not show significantly higher level of LOY compared with controls. However, in the latter cohort, the CD4 + lymphocytes and granulocytes were more frequently affected by LOY (*p* = 0.033 and *p* = 0.031, respectively). Although not proving direct evidence of causality, the observation that prostate cancer and Alzheimer’s disease patients were primarily affected with LOY in different types of immune cells would support a disease-specific link. This suggests that associations between LOY and disease cannot solely be attributed to a confounding age-dependent process of genomic instability, which would be expected to equally affect different types of leukocytes. Our results are thus compatible with both hypotheses presented in the introduction regarding aetiology of LOY and disease; accumulation of LOY in leukocytes as a barometer of genomic instability in somatic cells, as well as increasing risk for different types of disease when specific types of immune cells are affected with LOY.

## Discussion

Our study shows associations between LOY and dysregulation of autosomal gene expression in leukocytes, providing two major insights. First, it highlights a surprisingly broad molecular role for the Y chromosome, which is often considered a “genetic wasteland” with limited involvement in the aetiology of complex traits and diseases. The expression of *SRY* on the Y chromosome is required in mammals to override the development of the default female sex. The mammalian sex chromosomes evolved from a pair of autosomes over the past 300 million years and the Y chromosome has been remarkably stable over the past 25 million years [40, 41]. Characterization of human chromosome Y has, however, been limited by the challenges of sequencing the highly repetitive regions as well as assaying its sequence variation for testing in large population studies. Mosaic loss of chromosome Y in hematopoietic cells was long considered as a neutral event. However, this view has been challenged by recent epidemiological studies describing associations between LOY in blood cells and increased risk for various disease [6–15]. Furthermore, results from serially studied subjects found an association between LOY and clonal expansions in peripheral blood, with the frequency of LOY typically increasing in frequency over time in aging men [21]. However, some subjects showed other longitudinal patterns, suggesting that mechanisms such as age-related cellular senescence might be associated with recovery from LOY over time. Moreover, the co-occurrence of LOY and somatic mutations in genes linked with clonal hematopoiesis of indeterminate potential (CHIP) has been described in bone marrow samples from patients diagnosed with hematological malignancies [42]. Hence, the observed clonal expansion of LOY in peripheral blood might be related to such co-existence of LOY and CHIP mutations in progenitor cells and further studies are needed to understand their relative contribution and implications for clonal hematopoiesis and disease risk. Furthermore, two recent papers further highlight the importance of normal expression of chromosome Y genes in the context of risk for cancer and Alzheimer’s disease in men. By estimating the extreme down-regulation of chromosome Y (EDY) using transcriptomic data from 19 studies, it was found that EDY in blood cells was clearly associated with cancer risk (OR = 3.66) as well as greater EDY in tumor samples compared with normal tissue (OR = 8.33) [43]. A similar study that performed EDY analyses of transcriptomic data from five Alzheimer’s disease case–control studies found that the normal expression of chromosome Y genes (i.e., avoidance of EDY) in the brain could be associated with protection from developing this disease in aging men [44]. The results reported here, indicating that LOY is associated with dysregulated expression of ~ 500 autosomal genes, further support the notion that this chromosome may have a broader relevance in regulation of the transcriptome and associated risk for disease, than is currently appreciated.

Furthermore, we describe here associations indicating that patients with Alzheimer’s disease might primarily be affected with LOY in NK cells, while men diagnosed with prostate cancer appears to more frequently display LOY in CD4 + T cells and granulocytes. These observations suggest a cellular specificity of LOY that would support a disease-specific link, which needs to be replicated in additional cohorts. A cellular specificity of LOY with increased risk for different types of disease, together with the observation that the fraction of LATE genes within specific cell types was substantially larger than the fraction of LATE genes shared between different subsets of leukocytes (Supplementary Fig. 7), suggests that LOY might have pleiotropic effects. Pleiotropy is defined as an effect of one feature at the genetic level to multiple characteristics at the phenotypic level [45]. Considering the large number of epidemiological associations between LOY in blood and various disease outcomes, the LOY-pleiotropy might be a plausible concept. Our results are also in agreement with three experimental studies in mice, which support the potential cell type specific effects that Y chromosome may have on leukocyte populations and disease [46–49].

Second, our study highlights altered immune cell function that could conceivably link LOY in immune cells directly to disease mechanism(s). The combined number of LATE genes derived from analyses of various cells is surprisingly large, which suggests that a loss of ~ 2% of the male haploid genome (via LOY) could have an impact on cellular homeostasis. Enrichment analyses showed that LATE genes are important for a range of physiological functions (Supplementary Table 3) including many normal functions of the immune system. One of the LATE genes that showed the strongest downregulation was the autosomal gene *LAG3* (Fig. 2). The LAG3 protein is a cell surface molecule that functions as an immune checkpoint receptor by binding its main ligand MHC class II with higher affinity than CD4. Cellular proliferation and immune cell activation is regulated by a balance between CD4/LAG3, in a similar fashion to the CTLA4 and PD1 immune checkpoints, where *LAG3* expression suppresses cell activity [50, 51]. The observed low expression of *LAG3* in LOY cells might disrupt the CD4/LAG3 balance by releasing one of the breaks of immune cell activity. Furthermore, it is noteworthy that the immune checkpoint PD-1 signaling pathway is one of the top hits in the enrichment analysis (Supplementary Table 3).

Furthermore, the genes *LY6E* and *CD99* showed LATE by both RNAseq and scRNAseq in NK cells and monocytes (Fig. 2). The protein product of *CD99* is a cell surface glycoprotein involved in processes such as leukocyte migration, cell adhesion and apoptosis; e.g., by functioning as a diapedesis-mediating receptor central for migration of monocytes through endothelial junctions [52, 53]. A lower expression of *CD99* in leukocytes with LOY might, therefore, mitigate extravasation and thus impair the recruitment and movement of leukocytes from circulation towards somatic tissues and sites of disease. Furthermore, the *LY6E* gene was upregulated as an effect of LOY and it has the potential to inhibit inflammatory cytokines and disrupt inflammatory cascades. A survey of more than 130 published clinical studies found that increased expression of *LY6E* is associated with poor survival outcome in multiple malignancies [54] and it has also been found to be important for drug resistance and tumor immune escape in breast cancer [55]. Among all autosomal LATE genes, the *IL1R2* gene showed the strongest downregulation in immune cells with LOY. This cytokine receptor is central for orchestrating inflammatory and immune responses by binding of ligands such as interleukin-1α (IL1A), interleukin-1β (IL1B), and interleukin 1 receptor antagonist (IL1Ra), preventing them from binding to other receptors [56]. Hence, a lower abundance of *IL1R2* in LOY cells could reduce the inhibiting effect of this receptor.

Additionally, several of the LATE genes we identified had diverse and well-described roles in other biological processes, notably *GABRR1*, *PRLR*, and *LEP*. *GABRR1* encodes a receptor for Gamma Aminobutyric Acid (GABA), the major inhibitory neurotransmitter in the brain. A recent study found these receptors were functionally expressed in human and mouse hematopoietic stem cells and megakaryocyte progenitors [57]. Overexpression of *GABRR1* in these cells led to increased rates of megakaryocyte and platelet differentiation. In contrast, we observed 1.26-fold (*p* = 7.9 × 10^–5^) increased expression of the receptor in NK cells lacking the Y chromosome, extending the biological role of this receptor in immune cell function. Sex hormone receptors have also been described to have functional effects on hematopoietic stem/progenitor cells [58] including the prolactin receptor (*PRLR*), which we see over-expressed in monocytes with LOY (Supplementary Table 2). We also observed over-expression of the hunger inhibiting hormone Leptin (LEP) in granulocytes with LOY, consistent with studies describing its lesser known roles in regulating innate immune response [59].

## Conclusions

As mentioned in the introduction, LOY is the most common post-zygotic mutation from analyses of bulk DNA from peripheral blood. The current study adds new information on this as all 29 men (median age 80 years, 64–94 years) studied by scRNAseq carried leukocytes without chromosome Y. Thus, LOY is more common than so far appreciated and the future analyses of single- or sorted-cells from various tissues will be instrumental for establishment of true frequency of this mutation as well as for identification of cell subtypes most often associated with disease. Lastly, we describe that LOY is associated with dysregulation of ~ 500 autosomal genes, for example, involved in immune functions but also with roles in other biological processes. The present study provide insights into the molecular role of the Y chromosome and bring forth clues on how its mosaic loss in leukocytes could be associated with increased risk for disease in affected men, which ought to stimulate further research.

### Supplementary Information

Below is the link to the electronic supplementary material.

Supplementary file1 (PDF 2535 KB)

Supplementary file2 (XLSX 141 KB)

Supplementary file3 (XLSX 469 KB)

Supplementary file4 (XLSX 81 KB)

Supplementary file5 (XLSX 76 KB)

## Data Availability

The datasets used and/or analyzed during the current study are available from the corresponding author on reasonable request.
